# Analysis of connexin expression during seizures induced by 4-aminopyridine in the rat hippocampus

**DOI:** 10.1186/s12929-015-0176-5

**Published:** 2015-08-14

**Authors:** Medina-Ceja Laura, Flores-Ponce Xóchitl, Santerre Anne, Morales-Villagrán Alberto

**Affiliations:** Laboratory of Neurophysiology and Neurochemistry, Department of Cellular and Molecular Biology, CUCBA,University of Guadalajara, Camino Ing. R. Padilla Sánchez 2100, Las Agujas, Nextipac, Zapopan, Jalisco Mexico; Laboratory of Molecular Biomarkers and Molecular Genetic, Department of Cellular and Molecular Biology, CUCBA, University of Guadalajara, Jalisco, Mexico

**Keywords:** 4-Aminopyridine, Connexins, EEG, Hippocampus, Seizures

## Abstract

**Background:**

In epilepsy, seizures are generated by abnormal synchronous activity in neurons. In the rat hippocampus (HIP), epileptiform activity has been found to be associated with gap junctions (GJs). GJs are formed by the combination of two hemichannels, each composed of six connexins. At low doses, the convulsive drug 4-aminopyridine (4-AP) produces epileptiform activity without affecting glutamate levels; therefore, GJs could participate in its effect. Based on this argument, in this study, the expression of Cx 32, Cx 36 and Cx 43 protein and mRNA in the HIP of rats treated with 4-AP was evaluated. The evaluation of connexins was carried out by chemifluorescent immunoassay, semiquantitative RT-PCR and immunofluorescence to detect the amount and distribution of connexins and of cellular markers in the HIP and dentate gyrus (DG) of animals treated with NaCl and 4-AP in the right entorhinal cortex. In these animals, convulsive behavior and EEG signals were analyzed.

**Results:**

The animals treated with 4-AP showed convulsive behavior and epileptiform activity 60 min after the administration. A significant increase in the protein expression of Cx 32, Cx 36 and Cx 43 was found in the HIP contralateral and ipsilateral to the site of 4-AP administration. A trend toward an increase in the mRNA of Cx 32 and Cx 43 was also found. An increase in the cellular density of Cx 32 and Cx 43 was found in the right HIP and DG, and an increase in the cellular density of oligodendrocytes in the DG and a decrease in the number of cells marked with NeuN were observed in the left HIP.

**Conclusions:**

Cx 32 and Cx 43 associated with oligodendrocytes and astrocytes had an important role in the first stages of seizures induced by 4-AP, whereas Cx36 localized to neurons could be associated with later stages. Additionally, these results contribute to our understanding of the role of connexins in acute seizures and allow us to direct our efforts to other new anticonvulsant strategies for seizure treatment.

## Background

Epilepsy is a neurological disease that affects 2-5 % of the population worldwide; 67 million people have epilepsy [1]. In epilepsy, recurrent seizures are generated by abnormal synchronous activity in a group of neurons [2]. Currently, the cellular mechanisms involved in the generation of epilepsy are not known in detail. *In vitro* studies in the rat hippocampus have found epileptiform activity in the absence of chemical synapses. This fact suggests that other mechanisms may be involved in epilepsy, such as electrotonic coupling between cells [3–5]. Electrotonic coupling is produced by gap junctions (GJs). GJs are intercellular connections formed by the addition of two hemichannels called connexons. Every connexon is formed by six structural transmembrane proteins, called connexins (Cxs). Cxs 26, 30, 32, 36, 43, and 47 are expressed in the rodent hippocampus [6, 7]. Astrocytes express Cxs 30, 43 and 26, oligodendrocytes express Cxs 29, 32 and 47, and neurons express Cxs 36 and 45 [8, 9]. The union of two connexons forms a channel through which ions and small molecules (<1000 Da; AMPc and IP3) pass bidirectionally [10, 11].

Several *in vitro* and *in vivo* studies have found a relation between GJs and epileptiform activity [12–14]. In this respect, GJ blockers, such as carbenoxolone, octanol and quinine, have an antiepileptic effect [15–20], whereas GJ openers, such as trimethylamine, facilitate epileptiform activity [17, 21]. Additionally, some authors have reported changes in the expression of Cxs 30, 32, 36 and 43 in seizure and epilepsy models [15–17, 22–27] as well as in patients with mesial temporal lobe epilepsy [28–31]. Nevertheless, the results of these studies are contradictory. There is evidence of the importance of Cxs in brain function. Animals in which different Cxs have been knocked out exhibit alterations in gamma oscillations [32] and myelination, neuronal hyperexcitability [33], and a reduction of calcium waves [28, 34]. Some Cxs knockout lines even exhibit epileptiform activity and changes in glutamatergic transmission in the hippocampus [35, 36]. There is electrical coupling between hippocampal cells and in the entorhinal cortex [37–39]. These limbic structures have a role in the hypersynchronization that underlies epileptiform activity, and these brain regions are important sites for seizure generation [12, 20, 40].

4-Aminopyridine is a convulsant drug that blocks potassium channels and prolongs the action potentials of neurons; this effect facilitates the nonspecific release of neurotransmitters such as glutamate in the hippocampus and striatum [41, 42]. Low doses of 4-AP have been observed to produce epileptiform activity without affecting glutamate levels [43, 44], suggesting that other mechanisms could be involved in seizure generation in this model, such as GJs. For this reason and because there have not been any studies that have addressed these issues, in the present study we evaluated protein and mRNA expression of Cxs 32, 36 and 43 in the hippocampus and dentate gyrus (DG) of freely moving rats treated with 4-AP in the right entorhinal cortex (rEC).

## Methods

### Animal surgery

Twenty-eight adult male Wistar rats (250–300 g in weight) were used in the present study. All rats were maintained in individual cages in a temperature-controlled room with a 12-h light/dark cycle, with *ad libitum* access to food and water. All experimental procedures were designed to minimize animal suffering and the total number of animals used. This protocol conformed to the Rules for Research in Health Matters (Mexican Official Norms NOM-062-ZOO-1999, NOM-033-ZOO-1995) and was approved by the local Animal Care Committee.

Rats were first anesthetized with isoflurane in 100 % O_2_ and then secured in a Stoelting stereotaxic frame with the incisor bar positioned at −3.3 mm. A stainless steel guide cannula (0.5 mm internal diameter) was implanted into each rat through a hole drilled in the skull, and it was positioned in the rEC at the following stereotaxic coordinates relative to bregma (rEC: AP, −8 mm; L, 4.6 mm; V, 4 mm). This cannula was used to insert an injection needle (V, 5 mm) that also served as a recording electrode. The entire surface of the cannula was insulated except for a 1 mm portion of its tip. Three stainless steel screws were attached to the skull, one above bregma and two above the cerebellum, which served as the ground and the reference electrodes, respectively. Three surface electrodes with the same characteristics as the electrodes described above were implanted into the skull over the right frontal cortex (rFC), the left frontal cortex (lFC) and the left occipital cortex (lOC). The guide cannula and surface electrode wires were attached to a socket connector and fixed to the skull with acrylic dental cement. This procedure was completed in animals from both the control and the experimental group to perform the EEG recordings as well as for the immunoassay tests. Animals used to evaluate mRNA and immunohistochemistry by fluorescence were implanted only with the guide cannula.

### Drug administration and EEG recording

After surgery, the animals were allowed to recover for 24–48 h and then divided into experimental groups and treated as follows: 3 control groups (one group with *n* = 4 and two groups with *n* = 3) received an injection of the vehicle alone (NaCl, 0.9 %), and 60 min after the injection the animals were sacrificed; and 3 experimental groups of animals (*n* = 6 per group) were injected with 10 nmol 4-AP and sacrificed similarly, 60 min after the injection.

4-AP (Sigma, MO, USA) was diluted in the appropriate concentration of NaCl to maintain iso-osmolarity. Both the vehicle and 4-AP solutions were injected locally into the rEC at a flow rate of 1.0 μl/min for 1 min (final volume: 1 μl) using a microsyringe mounted on a microinjection pump (WPI SP101, FL, USA).

The rats were placed in a containment unit, and the electrodes were connected to a cable fixed to a balanced arm. EEG activity was recorded using a Grass polygraph model 6 with a low-frequency filter at 1 Hz and a high frequency filter at 300 Hz and was sampled at 100 Hz/channel (four channels). Data were stored on a computer hard disk and analyzed with AcqKnowledge software from Biopac Systems MP150 (Biopac Systems, Inc., CA, USA). After recording basal activity for 30 min, vehicle or 4-AP (10 nmol) was administered. Then, the animals were observed continuously for 60 min, during which time EEG activity was recorded. The basal electrical activity of each group was analyzed, and the amplitude and frequency were averaged over a 5 min recording period. The data from the experimental groups were analyzed by measuring the amplitude of a single epileptiform discharge over a 3 min period of the recording. Amplitudes were sampled at different times after the drug administration. The frequency of the EEG epileptiform activity (the number of single epileptiform discharges during a 1 s seizure period) was averaged manually for 3 min for each of the different periods of time studied. Propagation of this epileptiform activity to other areas was also analyzed.

### Behavioral study

Animal behavior was scored by continuous observation before, during and after vehicle or drug administration, using a modified version of the Racine scale [19]. Briefly, behavior was scored as follows: 0, behavioral arrest (motionless), piloerection, excitement and rapid breathing; 1, movement of the mouth, lips, tongue and vibrissae as well as salivation; 2, head and eye clonus; 3, forelimb clonus, “wet dog shakes”; 4, clonic rearing; 5, clonic rearing with a loss of postural control and uncontrolled jumping.

### Synaptosome and glial membrane extraction

Connexin proteins are localized in nerve terminals (synaptosomes) and glial membranes; therefore, we used the method of Li and coworkers [45] to extract them. After 60 min of vehicle or 4-AP administration, animals from the control and experimental groups belonging to the first set of experiments were decapitated, and the right and left hippocampus were extracted and weighed. Next, the tissue was homogenized in cold PBS (pH 7.3; 0.12 M) containing 0.32 M sucrose, 1 mM NaHCO_3_, 1 M MgCl_2_ and 0.5 mM CaCl_2_ by applying 12–24 strokes (800 rpm; 50 % amplitude) with an ultrasonic tissue processor (Cole-Parmer, IL, USA). Then, the homogenized solution was centrifuged for 10 min (1400 X g). The supernatant was collected, and the nuclear precipitate was homogenized and centrifuged for 10 min (1400 X g); this last supernatant was combined with the previous supernatant, and they were centrifuged for 30 min (13800 X g). The precipitate obtained contained crude synaptosomes and mitochondria, and this precipitate was suspended in cold phosphate-buffered saline (PBS, 0.12 M; pH 7.3) to be used in the immunoassay.

### Immunoassay by chemifluorescence

This procedure was performed according to Hendrix and coworkers [45] with some modifications. Microplates of 96 wells with a solid support were used; the plates contained protein G in order to fix the primary antibody (Thermo Scientific, IL, USA). In every microplate, a primary antibody for Cx 32 (anti-mouse polyclonal antibody; Zymed, CA, USA), Cx 36 (anti-rabbit polyclonal antibody; Santa Cruz Biotechnology, CA, USA) and Cx 43 (anti-rabbit polyclonal antibody; Santa Cruz Biotechnology, CA, USA), diluted 1:500 with PBS (0.12 M, pH 7.3), was incubated overnight at 4 °C. After that, three washes with PBS were applied, each for 10 min with shaking. The microplates were then incubated with blocking solution (0.12 M PBS, pH 7.3; 0.01 % NaN_3_; and 1 % bovine serum albumin (BSA), pH 7.6) for 1 h at room temperature with shaking. Then, three 10 min washes with PBS containing Tween 20 (0.02 %) and NaN_3_ (0.01 %) and shaking were performed, followed by one 10 min wash with PBS and shaking at room temperature. Then, the synaptosome and glial membrane extracts in the microplates were incubated with diluted blocking solution for 10 min with shaking at room temperature. After that, the appropriate secondary antibody coupled to horseradish peroxidase (IgG anti-rabbit or IgG anti-mouse; Abcam, CA, USA), diluted 1:500 in blocking solution, was added to every microplate to incubate for 1 h. After that, three washes of 10 min each were performed with PBS containing Tween 20 (0.02 %) and NaN_3_ (0.01 %) and shaking. Subsequently, one wash of 10 min with only PBS was performed. Finally, the reaction mixture (Amplex-enhancer solution, 10-acetyl-3,7-dihydroxyphenoxazine (ADHP) and stable peroxidase (QuantaRed Enhanced Chemifluorescent HRP Substrate Kit; Thermo Scientific, IL, USA) was added to every microplate and incubated in the dark for 10 min at room temperature with shaking. The reaction was stopped with a QuantaRed stop solution (Thermo Scientific, IL, USA), which was added to every microplate for 15 s while shaking. Immediately, the chemifluorescence was read in each well of the microplates with a CCD detector (USB 4000, Ocean Optics) that contained two optic fibers. The excitation wavelength was 570 nm and the emitted light was captured at 585 nm.

### Semiquantitative RT-PCR of connexins

After 60 min of vehicle or 4-AP administration, animals from the control and experimental groups belonging to the second sets of experiments were decapitated, and the right and left hippocampi were extracted and weighed. Each hippocampus was preserved at −80 °C in 1 ml of Trizol RNA extraction reagent, as indicated by the manufacturer (Invitrogen, CA, USA). The defrosted hippocampus was homogenized, and approximately 50 mg of this material was used for each RNA extraction. To determine the integrity of the total RNA extracted, agarose gel electrophoresis was used, whereas the concentration and purity of the total RNA was probed using a spectrophotometer (LABOMED, CA, USA). cDNA was synthesized from 500 ng total RNA using oligo-dT_12–18_ and M-MLV reverse transcriptase as indicated by the manufacturer (Invitrogen, CA, USA). The cDNA was amplified with a variation of PCR called multiplex-PCR, in which two or more primers are used in the same reaction mixture. cDNA (2 μl) was added to the reaction mixture (23 μl), which contained: dNTP Mix, 0.4 mM; PCR buffer, 1X; MgCl_2,_ 4 mM; *Taq* DNA Polymerase, 2U final concentration (Invitrogen, CA, USA) and the different primers (forward and reverse) for Cx36 and Cx43, 0.4 μM. Another prepared reaction mixture was used for the addition of different primers (0.4 μM) (forward and reverse) for Cx32 and β-actin (constitutive gene). The conditions used for the multiplex RT-PCR were standardized to amplify both the Cx36/Cx43 and Cx32/ β-actin mixtures, as follows: initially 95 °C for 15 min for denaturation; 35 cycles of 95 °C for 50 s; 57 °C for 1 min for hybridization; 72 °C for 1 min for elongation; and a final incubation at 72 °C for 7 min. This procedure was carried out with a thermocycler (T100 Bio-Rad, CA, USA). The characteristics of the primers used (IDT, Inc, CA, USA) are shown in Table 1. Ten microliters of the PCR products were separated by electrophoresis. The gel was stained with ethidium bromide, and the results were documented using Kodak I.D. Image analysis software, Version 3.5 (New Haven CT, USA). The data were normalized to the constitutive gene β-actin. Each sample was tested in triplicate for each connexin and the constitutive gene.

**Table 1:** Characteristics of the primers used in the Semiquantitative RT-PCR of connexins

Gene	Primer sequence forward and reverse	Nucleotide position	NCBI data base ref	Expect product (bp)	Hybridization temperature (°C)
C × 32	5′-CGGCATCTGCATTATCCTCAAC-3′	643-664	BC078868.1	163	57
	5′-CAGCAGCTTGTTGATCTCATTCTG-3′	782-805			
C × 36	5′-GCAGAGAGAACGCCGGTACT-3′	424-443	Y16898	270	57
	5′-CTTGGACCTTGCTGCTGTGC-3′	660-679			
C × 43	5′-GATGAGGAAGGAAGAGAAG-3′	493-511	AY324140	620	57
	5′-CGCTAGCTTGCTTGTTGTAA-3′	1096-1105			
β-actin	5′-GCAAGAGAGGTATCCTGACC-3′	260-279	BC063166.1	318	57
	5′-CCCTCGTAGATGGGCACAGT-3′	565-584			

[26, 59]

### Perfusion and immunofluorescence

After 60 min of vehicle or 4-AP administration, animals from the control and experimental groups for the third set of experiments were anaesthetized with Nembutal (60 mg/kg, i.p.) and transcardially perfused with phosphate-buffered saline (PBS, 0.1 M, pH 7.4, 37 °C), followed by 4 % paraformaldehyde (in 0.1 M PBS, pH 7.4) containing 0.1 % glutaraldehyde. The animal’s brain was removed and post-fixed for 12 to 16 h at 4 °C. The brains were then mounted on a vibratome (WPI, FL, USA) and sliced coronally at a thickness of 30 μm. These sections were collected consecutively in separate wells of an incubation chamber containing PBS, and the immunofluorescence procedure was performed as described previously, with some modifications [22].

Antibodies directed against the Cxs were used, as follows: 32 (mouse monoclonal antiserum, Invitrogen, CA, USA), 36 (rabbit polyclonal antiserum, Invitrogen, CA, USA), and 43 (rabbit polyclonal antiserum, Invitrogen, CA, USA). Antibodies for the identification of neurons and glial cells were also used, as follows: NeuN (mouse monoclonal antiserum, Millipore, CA, USA), oligodendrocytes (mouse monoclonal antiserum, Millipore, CA, USA) and GFAP (rabbit polyclonal antiserum, Invitrogen, CA, USA).

Tissue sections were washed twice for 15 min in PBS (pH 7.4, 0.1 M), and then they were incubated for 30 min at room temperature with shaking in a blocking solution containing 10 % normal goat serum (NGS) and 0.1 % Triton X-100 in PBS. After three 15 min washes, the sections were incubated for 48 h at 4 °C with the primary antibodies (anti-Cx32, anti-Cx36 and anti-Cx43: 1:500; and Anti-NeuN, anti-oligodendrocytes and anti-GFAP: 1:1000), which were diluted in PBS containing 5 % NGS.

The sections were then washed five times with agitation for 15 min each in PBS and incubated with the secondary antibody with agitation for 2 h in darkness at room temperature. Cx36 and GFAP were detected using goat anti-rabbit IgG-Alexa 594 (1:1000, Invitrogen, OR, USA) in PBS containing 5 % NGS, whereas oligodendrocytes were detected using goat anti-mouse IgG-Alexa 594. Cx32 and NeuN were identified using goat anti-mouse IgG-Alexa 488 (1:1000, Invitrogen, OR, USA). Finally, Cx 43 was detected using goat anti-rabbit IgG-FITC (1:1000, Santa Cruz Biotechnology, CA, USA). Sections were washed four times in PBS (15 min each) with agitation in darkness before being mounted on glass slides and coverslipped using Vectashield (Vector Laboratories, Inc. Burlingame, CA, USA), to preserve fluorescence. The sections were examined by fluorescence microscopy (Olympus, U-LH100HG, Japan) and analyzed using ImageJ 1.48 software (NIH, MD, USA). Control sections were incubated without primary antibody or with the primary antibody replaced with NGS, and no fluorescence signal was observed in any of the control sections (data not shown).

### Cell counting and cellular density

The somas of neurons and astrocytes, marked with NeuN and GFAP, respectively, were counted in the CA1 and CA3 regions of the right and left hippocampus as well as in the DG region, using four slices per animal. With the 40x objective, three images per region were obtained and analyzed from each slice. The image had an area of 0.075 mm^2^; in this area, somas were counted until three areas per region were analyzed in each rat [46]. To measure cellular density in the slices stained for Cxs and oligodendrocytes, pixel intensity was measured in three fields; each field had an area of 0.075 mm^2^ and was obtained with the 40x objective. The analysis was performed in three fields from each studied region (CA1 and CA3 from the right and left hippocampus and the DG) obtained from each rat [46].

### Histological evaluation

To verify the location of the guide cannula in each experiment, some coronal brain sections (50 μm thick) were taken from rats previously perfused for immunofluorescence and that belonged to different experimental and control groups. These sections were stained with cresyl violet, and if the cannula was implanted incorrectly, the animals were excluded from the study.

### Data analysis

The EEG results and the immunoassay and immunofluorescence data are shown as the mean (± SEM), while the RT-PCR data are shown as the percentage change from the control group (mean ± SEM). Comparisons between the control and the experimental groups were performed by applying a Student’s *t*-test using the GraphPad Prism 6 statistical software package. Statistical significance was defined at p < 0.05.

## Results

### EEG activity in the control and experimental groups

**Fig. 1:**
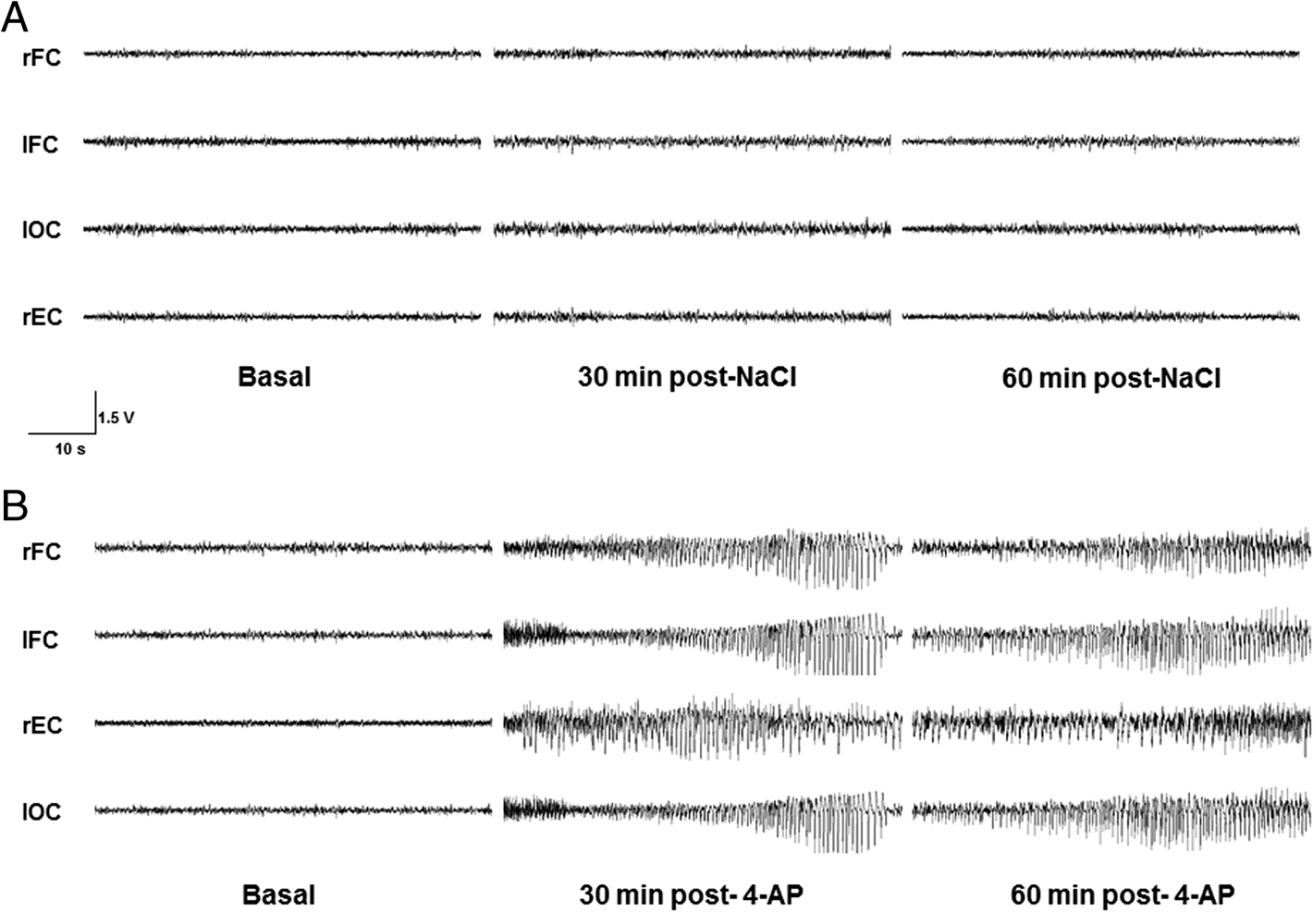
Representative EEG recordings from control (**a**) and experimental (**b**) rats in which NaCl (0.9 %) and 4-aminopyridine (4-AP, 10 nmol), respectively, were injected into the right entorhinal cortex (rEC). These recordings show the basal activity during and at different times after drug administration. No changes in EEG activity were observed in any of the regions studied (right and left frontal cortex, rFC and lFC, respectively; as well as in the left occipital cortex, lOC) after NaCl injection (**a**). The recordings show epileptiform activity of high amplitude and frequency after 4-AP injection (10 nmol) in all of the studied regions throughout the entire experiment (60 min)

**Fig. 2:**
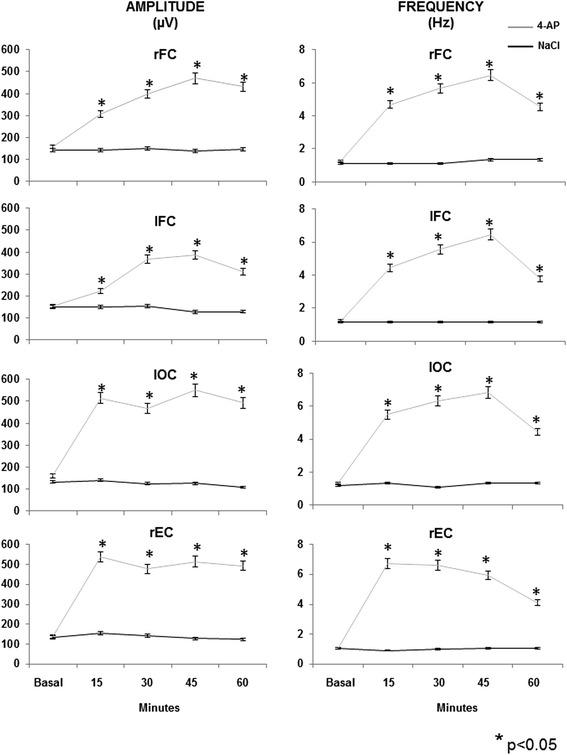
The graphs represent the mean (±SEM) of the different EEG amplitude and frequency parameters in the right entorhinal cortex (rEC), right and left frontal cortex (rFC and lFC, respectively) and left occipital cortex (lOC) of the control (NaCl, 0.9 %) and experimental (4-aminopyridine, 4-AP, 10 nmol) groups during basal activity and at different times after drug administration. Symbols represent: *p< 0.05 vs. control group

The basal EEG activity of the control animals that received NaCl and 4-AP was characterized by physiological slow waves of low amplitude and frequency (Figs. 1a, b and 2). The group of rats that received an injection of 4-AP into the rEC developed epileptiform activity that was initially characterized by hypersynchronous activity, followed by trains of poly-spikes that increased in amplitude and frequency during the experiment. The trains almost immediately propagated to all of the regions studied (latency to the first epileptiform discharge: rEC, 109 ± 49 s; rFC, 129 ± 42 s; lFC, 262 ± 109 s; and lOC, 132 ± 41 s; Fig. 1). At the end of the experiment (60 min), a slight decrease in the amplitude and frequency of the epileptiform activity was observed (Fig. 2).

#### Behavior of the control and experimental groups

The saline-treated rats presented normal behavior that was characterized by periods of sleep, sniffing and grooming, whereas the 4-AP-treated rats received scores of 0, 1, 2 and 3 on the modified Racine scale in the first 30 min after 4-AP injection, and one rat received a score of 5 (Table 2); 60 min after 4-AP injection, only one rat received a rating of 1 according to the scale; the rest of the animals continued to be rated as 3 based on the scale. One 4-AP-treated rat did not necessarily follow the progression of the Racine Scale and exhibited behavior that was rated as 3 on the scale without having received a scale rating of 2 (Table 2).

**Table 2:** Seizure behavior of rats injected with 4-AP (10 nmol, n = 6) at different times after 4-AP administration

Convulsive behavior after 4-AP injection				
RAT	Minutes			
	15	30	45	60
1	0/1	0/1/2/3	0/1/2/3	0/1/2/3
2	0/1/2/3	0/1/2/3	0/1/2	0/1
3	0/1/2	0/1/2/3	0/1/2/3	0/1/2/3
4	0/1/2	0/1/2/3	0/1/2/3	0/1/2/3
5	0/1	0/1/3	0/1/3	0/1/3
6	0/1/2/3	0/1/2/3/4/5	0/1/2/3/4/5	0/1/2/3

The values represent seizure behavior according to the modified Racine Scale [19]:0, behavioral arrest (motionless), piloerection, excitement and rapid breathing; 1, movement of the mouth, lips, tongue and vibrissae as well as salivation; 2, head and eye clonus; 3, forelimb clonus, “wet dog shakes”; 4, clonic rearing; 5, clonic rearing with a loss of postural control and uncontrolled jumping

#### Immunoassay and RT-PCR of connexins in the control and experimental groups

**Fig. 3:**
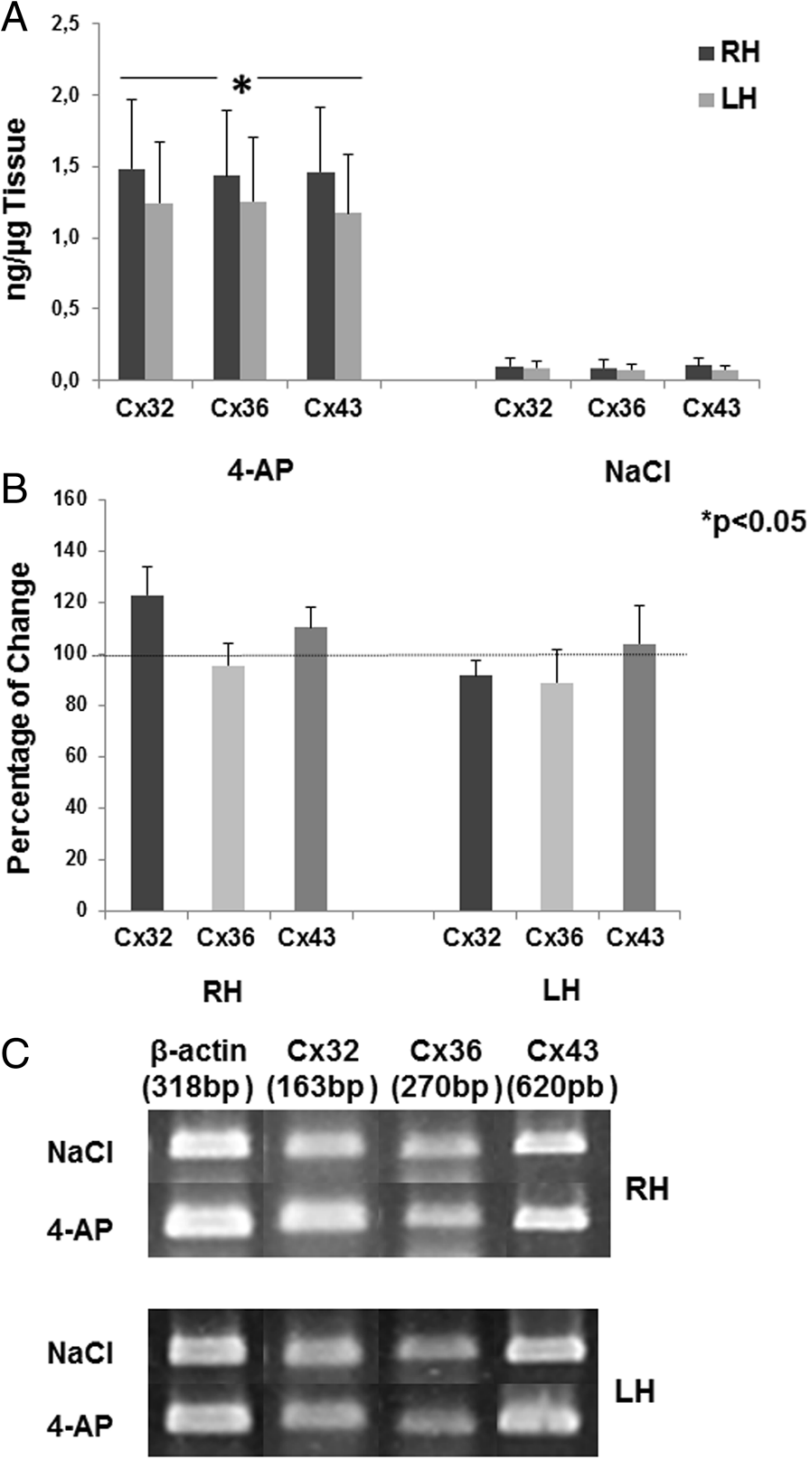
Panel **a** shows the mean (±SEM) amount of protein (ng/μg tissue) found in the right and left hippocampus (RH and LH, respectively) of connexins 32, 36 and 46 from control (NaCl, 0.9 %) and experimental rats (4-aminopyridine, 4-AP, 10 nmol). Panel **b** shows the percentage change with respect to the control group in the expression of the mRNA of different connexins in the RH and the LH. **c** representative images obtained from control (NaCl)- and experimental (4-AP)-treated rats, in which the expression of mRNA of different connexins (32, 36 and 43) was observed

Compared with the saline control group, the 4-AP-treated rats showed a significant increase in the protein levels of Cxs 32, 36 and 43 in the hippocampus (Fig. 3a). This increase was higher in the right hippocampus, which was ipsilateral to the administration of 4-AP into the rEC.

Analysis of the expression of the mRNA of the different Cxs showed a tendency toward increased Cx 32 and Cx 43 mRNA levels in the right hippocampus in animals treated with 4-AP compared with the control group (23 % and 10 %, respectively), but the increase was not significant. The rest of the Cxs were unchanged in both the right and left hippocampus (Fig. 3b, c).

#### Connexin immunofluorescence in the control and experimental groups

**Fig. 4:**
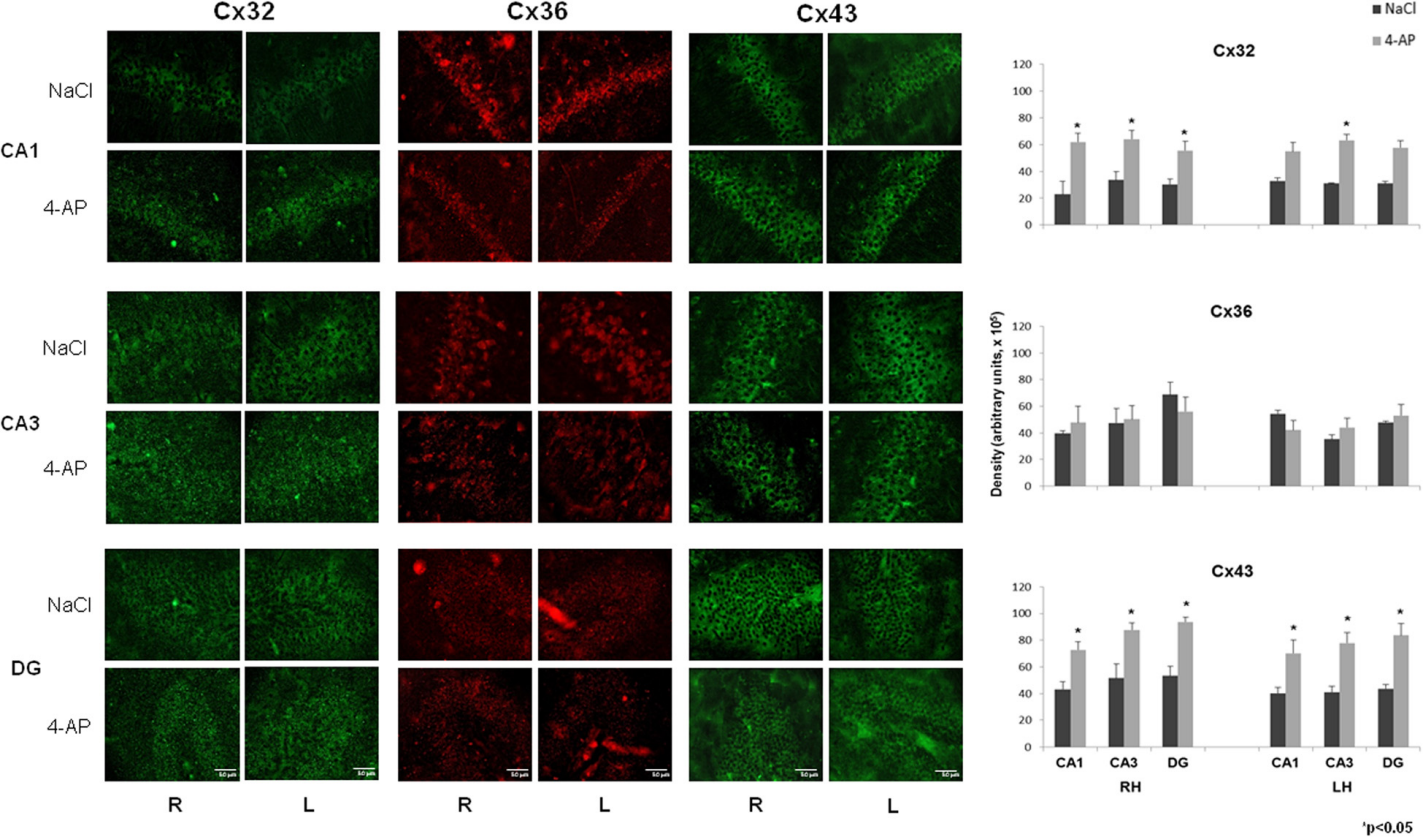
Representative images obtained with immunofluorescence to Cx 32, Cx 36 and Cx 43 in CA1 and CA3 of the right and left hippocampus (RH and LH, respectively) as well as in the dentate gyrus (DG) in the control (NaCl, 0.9 %) and experimental groups (4-AP, 10 nmol). Calibration bar, 50 μm. The right graphs represent the mean ± SEM of the cellular density for Cx 32-, Cx 36-, Cx 43-labelled cells in the three analyzed regions: CA1 and CA3 of the RH and LH, and the DG. Significant differences between the control and experimental groups were assessed with a Student’s *t*-test

**Fig. 5:**
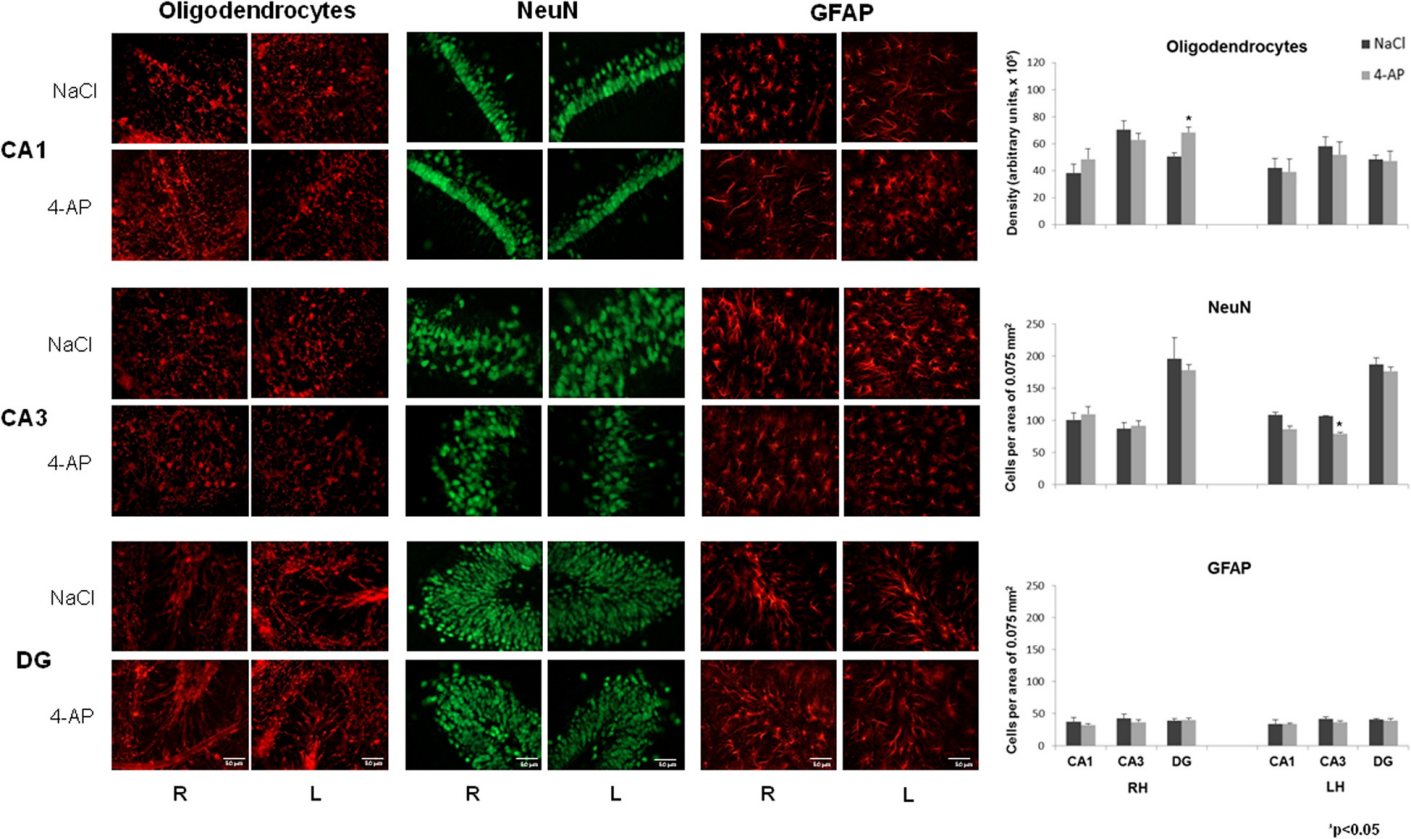
Representative images obtained with immunofluorescence to oligodendrocytes, NeuN and GFAP in CA3 and the dentate gyrus (DG) of the right and left hemisphere (RH and LH, respectively) in the control (NaCl, 0.9 %) and experimental groups (4-AP, 10 nmol). Calibration bar, 50 μm. The right graphs represent the mean ± SEM of the cellular density or cellular counting per area for oligodendrocytes, NeuN- and GFAP-labelled cells in the three analyzed regions: CA1 and CA3 of the RH and LH, and the DG. Significant differences between the control and experimental groups were assessed with a Student’s *t*-test

The 4-AP-treated animals showed an increase in the cellular density of Cx 32 and Cx 43 in CA1, CA3 and the DG of the right hippocampus compared with the control group (Fig. 4). In the LH, Cx 43 increased in all regions, whereas Cx 32 was increased only in CA3 with respect to the control group. Cx 36 did not change in any region analyzed in both the right and left hippocampus. When these data were compared with the corresponding cellular markers (Cx 32 with oligodendrocytes, Cx 36 with NeuN and Cx 43 with GFAP), only an increase in the cellular density of oligodendrocytes was found in the DG of the right hippocampus was found as well as a decrease in the number of cells marked with NeuN in the CA3 region of the left hippocsmpus (Fig. 5). The rest of the cellular markers remained unchanged in both the right and left hippocampus.

## Discussion

In the present study, local administration of 4-AP (10 nmols) into the rEC produced epileptiform activity, which was characterized by hypersynchronous activity, followed by trains of poly-spikes that propagated to all of the studied regions. This finding is consistent with previous studies in our laboratory [19, 20]. This epileptiform activity coincided with the convulsive behavior that was associated with a score of 3 on the modified Racine Scale [19].

An increase in the amount of Cx 32 protein and a tendency toward an increase in the amount of its mRNA were observed in the right hippocampus of animals treated with 4-AP (10 nmols, rEC; 60 min after its administration). These data suggest a possible relation between the synthesis of Cx 32 mRNA and protein during the convulsive event produced by 4-AP. The present results are consistent with previous studies in which an increase in Cx 32 (mRNA and protein) was observed in neocortex treated with 4-AP [15, 17] as well as *in vitro* preparations of the hippocampus treated with bicuculline [16]. However, during epileptogenesis, the connection between Cx 32 mRNA and protein could be found in specific regions associated with spontaneous seizure generation because an increase in Cx 32 mRNA has been observed in the neocortex and hippocampus in patients with Temporal Lobe Epilepsy [28, 31]. The present and previous results regarding Cx 32 expression demonstrate the important role of Cx 32 as a compensatory mechanism that blocks the convulsive process and epileptogenesis. Accordingly, some experimental evidence has suggested that Cx 32 expressed in oligodendrocytes could contribute to the buffering of K^+^, which is released during neuronal activity [47, 48], by enabling the sequential movement of these ions into the periaxonal cytoplasm from the soma of the oligodendrocyte through reflexive junctions. This phenomenon is extended to a network formed by oligodendrocyte-astrocyte junctions; in addition, Cx 32/Cx 43 double-knockout mice show clonic-tonic seizures and early mortality [49]. Although we found an increase in the cellular density of Cx 32 in the cells of the right hippocampus and DG that correspond to the ipsilateral region of 4-AP administration (rEC), this increase is also partially consistent with the increase in the cellular density of labeled oligodendrocytes in the rDG, considering that Cx 32 is mainly expressed in this type of glial cells. These results are consistent with previous studies [16, 23] in which an increase of Cx 32 was found in hippocampal slices treated with bicuculline (an antagonist of GABA_A_ receptors).

Additionally, we found a significant increase in Cx 43 protein and a trend toward up-regulation of its mRNA only in the right hippocampus, ipsilateral to the 4-AP administration. These results agree with previous studies in which an increase in Cx 43 protein was observed in hippocampal slices treated with pilocarpine and bicuculline [16, 50] as well with *in vivo* and *in vitro* preparations of the neocortex after 4-AP [15, 17] and bicuculline administration [16]. In addition, an increase in the mRNA of Cx 43 has been observed in the hippocampus of patients with TLE [29]. Therefore, the present data suggest that Cx 43 protein is synthesized and distributed in astrocytes under conditions of constant hyperexcitability for at least 60 min. At this respect, 4-AP increases glutamate levels at synaptic sites [42, 44] probably activating N-methyl-D-aspartate receptors (NMDA-R) which increase the intracellular Ca^2+^ and produce Ca2+/calmodulin dependent protein kinase II (CaM-KII) activation, this last protein phosphorylates Cx 43 and this effect facilitating coupling between astrocytes [51, 52] as well as it has been reported a potentiation of the gap junction dependent on postsynaptic NMDA-R [53, 54] and phosphorylation of Cx 43 occur following *in vitro* Co^2+−^induced seizures [55]; besides that 4-AP generates hyperexcitability also produces oxidative stress in cells [56] that could facilitates that more connexins reach the membrane due to a decrease in the endoplasmic reticulum associated degradation of connexins [57, 58]. Additionally, the increase in Cx 43 in astrocytes could facilitate and sustain the epileptiform activity in the hippocampus, as has been demonstrated indirectly in a model in which carbenoxolone administration blocked epileptiform activity 60 min after 4-AP administration [19]. In addition, the hemi-channels formed by Cx 43 can be opened after voltage changes that depolarize the membrane during epileptiform activity [59]. ATP and glutamate are also released from astrocytes through these hemi-channels, producing neuronal death, as has been observed in epileptic tissue [60]. Additionally, an increase in the cellular density of Cx 43 protein was observed in cells of the hippocampus and DG from the right and left hemispheres in this model of 4-AP; however, we did not find any significant changes in the number of astrocytes in these same regions. This could be due to the process of glial scar formation over time because it usually takes several days to see active astrocytes after seizures [61]. In the present study, the experimental animals were sacrificed one hour after 4-AP administration, when an epileptiform pattern was observed.

We also did not find changes in Cx 36 in neurons from the hippocampus and DG. In addition, the number of neurons labeled with NeuN did not differ significantly between the experimental and the control animals in all of the studied regions except CA3. These data contrast with the increase in Cx 36 protein observed in the hippocampus of the experimental animals of this study. However, we did not find any changes in Cx 36 mRNA, which in consistent with previous studies in which no changes in Cx 36 mRNA were observed in hippocampal slices exposed to bicuculline [16] or in pilocarpine model during the acute phase when epileptiform activity was observed [62]; however, another study showed an increase in Cx 36 mRNA 60 min after the first seizure [17]. Finally, a third study reported a significant decrease in Cx 36 mRNA and protein in the hippocampus of rats treated with kainic acid and kindling [22]. This discrepancy in results could be due to differences in the animals (ages, strains), the model of seizure induction, the time of treatment, the duration of seizures before the animals were sacrificed and the regions analyzed in each study. However, our results showed an increase in Cx 36 protein only, that could be explained by a compensation of the degraded pool of Cx 36 affected by seizures effects (depolarization, increases in intracellular Ca^2+^ and oxidative stress in cells) considering the rapid half-life of Cx 36, which is on the order of tens of minutes [63], in contrast, the average half-lives of Cx 32 and Cx 43 that are approximately 1–4 h [64, 65].

Also of interest is the influence of the external and internal factors that regulate the opening and closing of the gap junctions through interactions with different Cxs. Regarding this process, some excitatory and inhibitory amino acids regulate the electrical coupling between neurons and astrocytes through second messenger signaling systems and kinase proteins [66]. In the present study, 4-AP slowed membrane repolarization and facilitated the release of neurotransmitters, especially glutamate; this neurotransmitter has been shown to influence the conductance of GJs in the hippocampus and to generate seizures [42]. Other regulating factors include pH and the intracellular calcium concentration; increases in both of these factors decrease the conductance of GJs. In the present study, the increase of glutamate produced by 4-AP resulted in the hyperactivation of ionotropic receptors that also modify the calcium concentration and pH of postsynaptic neurons, producing hyperexcitability and modification of the conductance of GJs. In addition, the alkalization mediated by potassium produced during the first seizure events increases electrotonic coupling; later, when it produces acidification at the end of the seizures, this coupling is reduced [67]. Based on these arguments, it is possible to think that the function of GJs formed by Cxs is altered even if the expression of the Cxs does not change significantly.

## Conclusion

4-AP administration in the rEC produced epileptiform activity associated with a score of 3 on the modified Racine Scale. Sixty minutes after the establishment of this epileptiform pattern, we observed an increase in the protein expression of Cx 32 and Cx 43, which was associated with a trend toward an increase in their mRNA as well as a trend toward an increase in the principal marker of oligodendrocytes in the right DG; these Cxs were also expressed in principal cells of the hippocampus and DG. Therefore, we suggest that the Cxs particularly associated with glial cells such as oligodendrocytes and astrocytes have an important role in the first stages of seizures induced by 4-AP. It is likely that the participation of Cx 36 in neurons is associated with the later stages of the acute convulsive process induced by 4-AP. These results contribute to our understanding of the role of Cxs in acute seizures and allow us to direct our efforts to other new anticonvulsant strategies for seizure treatment.
